# Recurrent single-CpG methylation differences and incomplete segregation in familial type 1 diabetes

**DOI:** 10.3389/fimmu.2026.1873945

**Published:** 2026-09-28

**Authors:** Mohammed Dashti, Md Zubbair Malik, Rasheeba Nizam, Fahd Al-Mulla

**Affiliations:** Translational Research Department, Dasman Diabetes Institute, Kuwait City, Kuwait

**Keywords:** DNA Methylation, epigenetics, familial segregation, HLA, type 1 diabetes

## Abstract

**Background:**

Type 1 diabetes (T1D) clusters within families, yet shared genetic susceptibility does not fully explain discordance among relatives. We investigated whether single CpG methylation differences recur independently across multiplex T1D families and whether methylation at these loci segregates with T1D status or relates to HLA class II risk haplotype dosage.

**Methods:**

Seventeen participants from three multiplex Arab families, including nine with T1D, underwent targeted bisulfite sequencing. Data from two sequencing runs were combined. Each pedigree was analysed independently. Unadjusted within-family analyses defined the cross-family recurrent set, requiring q<0.01, |Δβ|≥25% and concordant direction in all three families; age- and sex-adjusted analyses were then used to prioritise recurrent candidates. Candidate loci underwent pyrosequencing; public T1D transcriptomic datasets provided context for annotated genes. HLA genotyping determined risk haplotype dosage, and donor-level models examined associations with pyrosequencing methylation while accounting for family structure.

**Results:**

Unadjusted within-family analyses identified 22 recurrent CpGs with concordant methylation direction across the three pedigrees. Six of these retained the prespecified criteria after adjustment for age and sex and were prioritised for subsequent analyses. These mapped to *MYRIP*, *DLGAP2*, *ULK4*, *MTCL1*, *LINC00701* and *CCNG1*. Pyrosequencing of *MYRIP*, *ULK4* and *MTCL1* revealed distinct methylation distributions, with affected and unaffected relatives occupying overlapping states or ranges, consistent with incomplete within-family segregation. HLA risk haplotype dosage showed a gradient in T1D occurrence across the families. *MYRIP* methylation was nominally lower in participants with T1D in the unadjusted donor-level analysis, but did not survive multiple testing correction. In exploratory adjusted donor-level models, higher HLA risk dosage was associated with lower *MYRIP* methylation.

**Conclusion:**

These findings identify recurrent single-CpG methylation differences across multiplex T1D pedigrees but show incomplete individual-level segregation with disease. The six prioritised loci should therefore be considered exploratory methylation candidates whose temporal relationship with T1D and reproducibility require evaluation in larger independent and longitudinal family cohorts.

## Introduction

Type 1 diabetes (T1D) results from immune-mediated destruction of pancreatic β-cells and requires lifelong exogenous insulin therapy. Childhood incidence has risen over recent decades, and the Arabian Gulf region carries a disproportionate burden: in Kuwait, the rate reached 40.9 per 100,000 per year by 2011–2013, more than double the figure recorded in the early 1990s (1). High background consanguinity may increase autozygosity and enrich familial risk variants, potentially accentuating disease clustering in multiplex families (2, 3). The genetic architecture of T1D is well characterised: the HLA region accounts for approximately 50% of heritable susceptibility, with additional contributions from non-HLA loci identified by genome-wide association studies (4). Yet monozygotic twin concordance is 35 to 65% even with extended follow-up (5), and affected and unaffected siblings within multiplex families often carry identical high-risk HLA haplotypes. This within-family discordance points to regulatory mechanisms that modulate disease risk against a shared genetic background.

T1D is therefore best understood as a multifactorial disease in which inherited susceptibility, environmental exposures, and epigenetic regulation converge to drive immune dysregulation, β-cell stress, and islet autoimmunity (**Figure 1**). HLA class II haplotypes (DR3-DQ2 and DR4-DQ8) are the strongest inherited risk factors but do not fully explain familial or twin discordance (4). HLA allele and haplotype distributions show population-specific variation in Kuwait and across Arab populations (6, 7). Environmental exposures, including enteroviral infection, gut microbiome composition, infant feeding, vitamin D status, and maternal metabolic state, have been proposed as modifiers of islet autoimmunity, with variable strength of evidence (8). Epigenetic mechanisms (DNA methylation, histone modifications, non-coding RNAs) provide a regulatory interface through which genetic background and environmental exposures may influence gene expression without altering DNA sequence (9).

**Figure 1:**
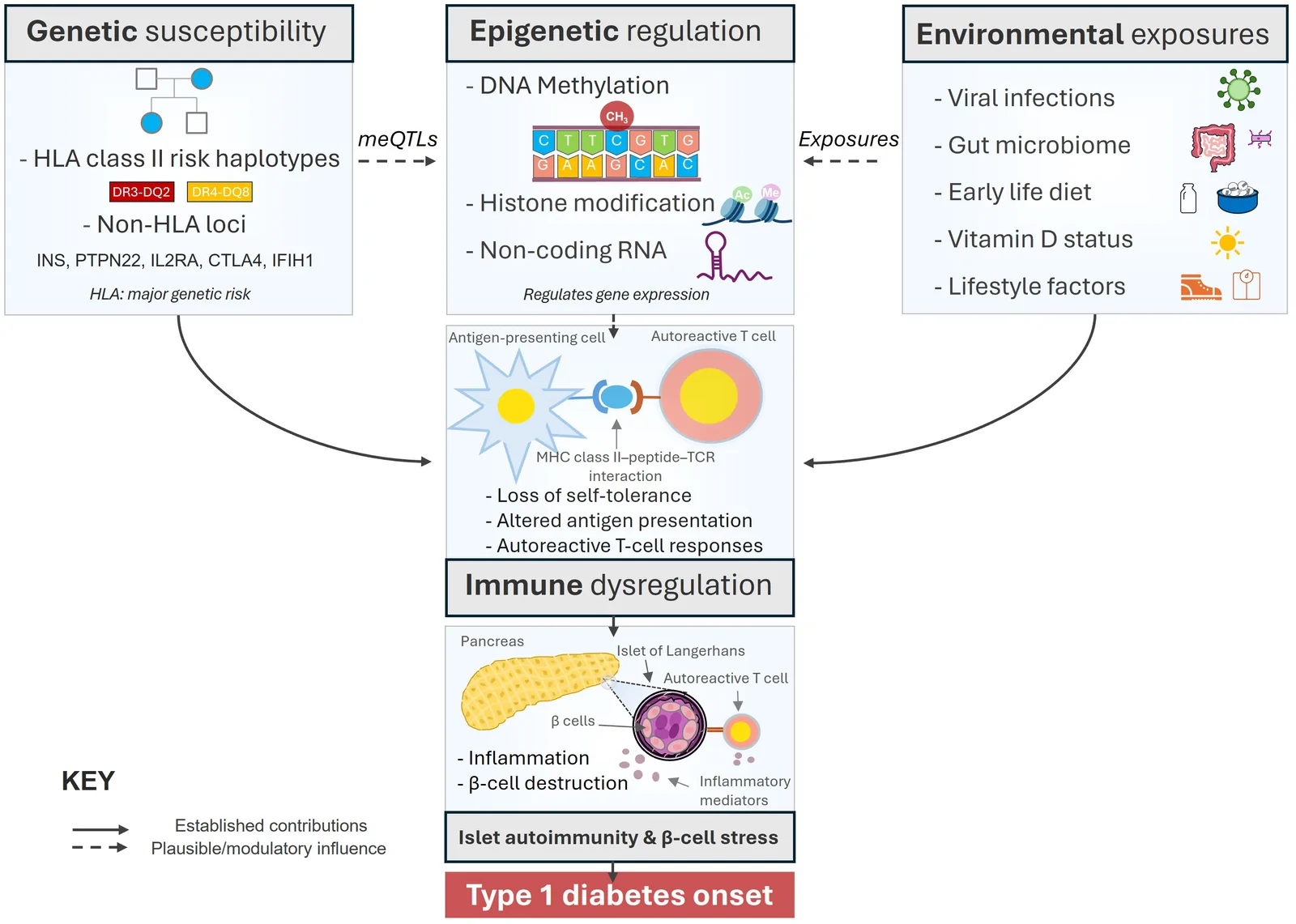
Conceptual framework linking genetic susceptibility, epigenetic regulation, and environmental exposures in T1D pathogenesis. Genetic susceptibility, environmental exposures and epigenetic regulation converge on immune dysregulation, β-cell stress, islet autoimmunity and clinical disease onset. The central immune panel depicts antigen presentation by an antigen-presenting cell to an autoreactive T cell through an MHC class II–peptide–T-cell receptor interaction. The lower panel depicts the pancreas, an islet of Langerhans, β cells, an autoreactive T cell and inflammatory mediators involved in islet inflammation and β-cell destruction. The dashed arrow from genetic susceptibility to epigenetic regulation denotes methylation quantitative trait locus effects, whereas the dashed arrow from environmental exposures denotes exposure-associated epigenetic modulation. Solid arrows indicate established contributions, and dashed arrows indicate plausible or modulatory influences.

DNA methylation is particularly relevant because it can reflect genotype, immune activation, environmental exposure, and cellular history, and persists through cell divisions, preserving regulatory information over time. Prior epigenome-wide and targeted methylation studies in T1D are summarised in **Supplementary Table 1** (10–22). EWAS have identified differentially methylated CpGs in immune cell populations from affected individuals, with *TXNIP* hypomethylation (cg19693031) appearing consistently across cohorts (23, 24); however, this signal has also been reported in type 2 diabetes and sustained hyperglycaemia (25), suggesting a glycaemia-related rather than disease-specific effect. More informative for disease susceptibility are studies showing that methylation differences can precede clinical disease. Rakyan et al. (26) identified methylation variable positions in individuals who later developed T1D, including normoglycaemic individuals with islet autoantibodies. Johnson et al. (27) reported methylation differences from birth through pre-autoimmunity and pre-diagnosis in the DAISY cohort. Starskaia et al. (22) extended this evidence to purified T cell subsets, including changes at the *IRF5* promoter in CD4+ T cells before seroconversion. Ott et al. (28) reported differential methylation at over 1,600 CpG sites in offspring of mothers with T1D, including loci within the MHC region and at 15 established T1D susceptibility genes, with a methylation propensity score predicting islet autoimmunity even in offspring of unaffected mothers. These studies support a temporal relationship between methylation and T1D susceptibility, but do not establish whether recurrent methylation states segregate within multiplex families.

Two limitations in the existing literature are relevant to the present analysis. First, most T1D methylation studies have used Illumina array platforms (HumanMethylation450, EPIC v1, EPIC v2), which interrogate a predefined subset of the approximately 28 million CpG sites in the human genome (29). Array coverage favours promoters and CpG islands, with sparser representation of intronic, shore, enhancer, and intergenic regulatory regions, despite their potential relevance to context-dependent gene regulation (30–32). Targeted bisulfite sequencing can capture a broader CpG space, including enhancer and shore regions underrepresented on arrays (33). Second, most studies have adopted case-control designs in unrelated populations or pooled group comparisons; these approaches identify disease-associated methylation differences but cannot directly test whether a methylation state segregates with disease within families. Multiplex families offer a more constrained framework because affected and unaffected relatives share a genetic background and early developmental context. Extending within-family analysis across independent kindreds and intersecting concordant signals provides additional confidence beyond any single comparison.

In our previous study of three multiplex Arab families from Kuwait, targeted bisulfite sequencing with the TruSeq Methyl Capture EPIC kit was used to compare methylation between pooled groups of affected and unaffected individuals (34). That design identified differentially methylated genes at the group level, but the coverage depth from a single sequencing run limited the number of CpGs that could be evaluated consistently within each family. We therefore sought to identify single-CpG methylation differences that recur independently across three multiplex T1D pedigrees and to determine whether methylation at these loci shows within-family segregation with T1D status or is related to HLA class II risk-haplotype dosage.

In the present study, data from two sequencing runs of the same indexed libraries were combined to increase observed read coverage and expand the CpG set available for within-family analysis. Each family was first analysed without participant-level covariates to define the unadjusted cross-family overlap and was then reanalysed with adjustment for age at recruitment and sex. CpGs were retained when they met the statistical and effect-size criteria in all three families and showed concordant methylation direction. The six loci retaining the recurrence criteria after age and sex adjustment were prioritised for subsequent pyrosequencing assessment to examine individual-level methylation patterns and within-family segregation with T1D status. HLA class II genotyping was used to determine risk-haplotype dosage and to examine its relationship with methylation and disease status. Public T1D transcriptomic datasets were used to assess whether genes annotated to the recurrent loci were differentially expressed in blood-derived or pancreatic datasets and to provide biological context.

## Materials and methods

### Study cohort

Three multiplex Arab families, designated A, B and C, with familial aggregation of type 1 diabetes (T1D) were recruited from the Dasman Diabetes Institute, Kuwait. Family A included dizygotic twins concordant for T1D and an additional affected sibling. Family B included an affected father, an unaffected mother, three affected offspring including monozygotic twins concordant for T1D, and two unaffected offspring. Family C consisted of affected non-twinned siblings and unaffected parents. One pedigree had a documented consanguineous parental relationship; the other two did not. Participant ages ranged from 6 to 46 years.

T1D diagnosis was confirmed according to World Health Organization criteria (35). Unaffected relatives were normoglycaemic at recruitment, based on glycated haemoglobin (HbA1c) and plasma glucose measurements. Written informed consent was obtained from all adult participants and from the parents or legal guardians of participants younger than 18 years. The study was approved by the Ethical Review Committee of the Dasman Diabetes Institute (RA-2014-024) and conducted in accordance with the Declaration of Helsinki.

### HLA genotyping

Whole-exome sequencing data were available for all 17 participants. The research-grade exome data were generated using 100-bp paired-end sequencing, with overall exome coverage ranging from 42× to 76×, as described previously (36). Classical HLA class II genotypes at *DRB1*, *DQA1* and *DQB1* were inferred at two-field resolution using HLA-HD v1.7.0 (37) with the IPD-IMGT/HLA reference database v3.56 (38). HLA-HD was selected based on its performance in our previous benchmarking of NGS-based HLA inference from whole-exome data (36). HLA genotypes for affected participants had been reported previously (34); the present analysis extended HLA assignment to the remaining family members using the same approach.

Clinical-grade HLA class II typing was available for 13 participants, including both parents in each pedigree and eight of the nine participants with T1D. Genomic DNA from whole blood was extracted using the QIAamp DNA Blood Mini Kit (Qiagen) and typed using the Omixon Holotype HLA v3 kit (Omixon, Hungary). The assay uses locus-specific long-range PCR followed by enzymatic fragmentation, end repair, adenylation and index ligation, with sequencing on the Illumina MiSeq platform. Exome-derived HLA calls were compared with the clinical-grade results at first-field and second-field resolution in participants assessed by both methods. One participant had duplicate clinical-grade measurements. Discrepant second-field calls were additionally evaluated for whether they altered HLA risk-haplotype classification.

HLA haplotypes were reconstructed by Mendelian transmission within each pedigree rather than by statistical phasing. Each participant was then classified according to HLA class II risk-haplotype dosage as carrying zero, one or two high-risk haplotypes (**Supplementary Table 2**). The two-risk category included both DR3-DQ2 homozygous and DR3-DQ2/DR4-DQ8 compound-heterozygous genotypes. DR3-DQ2 was defined as DRB1*03:01-DQA1*05:01-DQB1*02:01. High-risk DR4-DQ8 was restricted to DRB1*04:01, DRB1*04:02, DRB1*04:04 or DRB1*04:05 paired with DQB1*03:02 (39). The DRB1*04:06-DQA1*03:03-DQB1*04:02 haplotype present in this cohort was therefore classified as non-risk.

### Assessment of local genetic variation

Whole-exome gVCFs from all 17 participants were examined at the hg19 coordinates of the six prioritised CpGs and within 500 bp on either side of each site. The underlying WES data for all participants were processed using the same GRCh37-based BWA, SAMtools/Picard and GATK variant-calling workflow described previously (34). At each CpG coordinate, gVCF reference blocks and read depth were inspected to determine whether the position was informatively covered. Explicit SNP and indel records were also queried within each surrounding interval. A reference genotype was not assigned when sequence coverage at the CpG was absent.

### DNA extraction and bisulfite sequencing

Genomic DNA was extracted from peripheral blood mononuclear cells (PBMCs) using the QIAamp DNA Blood Mini Kit (Qiagen). Bisulfite conversion was performed using the EZ DNA Methylation-Lightning Kit (Zymo Research). Targeted methylation libraries were prepared using the Illumina TruSeq Methyl Capture EPIC Library Prep Kit, which enriches approximately 3.3 million CpG sites across CpG islands, promoters, enhancers and other regulatory regions. Libraries were sequenced on an Illumina HiSeq 2500 platform. Bisulfite conversion efficiency was assessed from methylation in non-CpG CHH context, and mapping efficiency was recorded for each sample.

### Coverage optimisation by repeated sequencing of the same libraries

The same indexed TruSeq Methyl Capture EPIC libraries were sequenced in two separate sequencing runs on the Illumina HiSeq 2500 using different loading concentrations to improve cluster density and sequencing yield. The second run involved resequencing of the existing indexed libraries; no additional bisulfite conversion, target capture, library preparation or PCR amplification was performed. Sixteen of the 17 participant libraries were resequenced. Sample I_2_C was not included in the second run because its first-run coverage was considered sufficient.

For participants represented in both runs, reads from the two sequencing datasets were combined before methylation processing to increase observed locus-specific read depth and the number of CpGs meeting the prespecified coverage requirement across family members. The first-run data for I_2_C were retained unchanged in the combined dataset. Sequencing depth and CpG yield for the individual runs and the merged dataset are reported in **Tables 1**, **2**.

**Table 1:** Average sequencing coverage (x) per sample across the first run, second run, and combined dataset.

Sample	First run (x)	Second run (x)	Combined (x)
I_1_A	10.5	9.6	19.7
I_2_A	10.9	9.4	19.9
II_1_A	7.8	7.1	14.4
II_2_A	7.4	6.4	14.1
II_3_A	7.7	6.8	14.1
II_4_A	8.3	7.3	15.2
I_1_B	12.8	12.3	24.9
I_2_B	9.5	9.1	18.2
II_1_B	10.2	9.9	19.8
II_2_B	9.9	9.6	19.2
II_3_B	11.8	10.1	21.6
II_4_B	11.6	10.7	22.0
II_5_B	13.7	12.5	25.9
I_1_C	8.4	7.2	15.2
I_2_C	17.7	–	17.7
II_1_C	13.3	11.4	24.4
II_2_C	8.9	7.6	16.0
**Average**	**10.6**	**9.2**	**19.0**

– denotes sample not sequenced in the second run due to sufficient coverage in the first run. Run-specific and combined mean coverages were calculated over the CpG sets retained in each respective dataset and are therefore not additive.Bold values indicate the average sequencing coverage across all samples for each dataset (first run, second run, and combined dataset).

**Table 2:** Number of analysable CpG sites passing coverage criteria in each sequencing run and in the combined dataset.

Analysable CpG sites	First run	Second run	Combined
Single CpG sites (total)	85,922	45,727	630,081

### Bioinformatic analysis of DNA methylation

For participants represented in both sequencing runs, paired-end FASTQ files were concatenated by read direction, with forward reads combined with forward reads and reverse reads combined with reverse reads. The resulting paired-end datasets were processed as single combined sequencing datasets. Read quality was assessed using FastQC v0.11.9 (Babraham Bioinformatics, UK). Low-quality sequence and the first six bases of both forward and reverse reads were removed using Trim Galore! v0.3.3, with Cutadapt v1.14 (40). Trimmed reads were aligned to the hg19 human reference genome using Bismark v0.22.3 (41) with Bowtie2 v2.3.5.1 as the underlying aligner. hg19 was retained because the TruSeq Methyl Capture EPIC target coordinates are defined in this genome build. The first-run dataset from I_2_C was processed using the same workflow.

Coordinate-based deduplication was not applied in the primary analysis because the assay uses targeted enrichment and independently captured fragments may share genomic coordinates. Deduplication was examined separately as a processing sensitivity analysis using deduplicate_bismark in Bismark v0.22.3. The deduplicated alignments were subsequently processed using the same methylKit filtering procedures and the same unadjusted within-family differential methylation model used for the corresponding primary analysis.

Methylation calling and downstream processing were performed using methylKit v1.12.0 (42) in R v3.6.1. Bismark paired-end alignments were imported using methylKit::processBismarkAln in CpG context with a minimum coverage of 10 reads, a minimum base quality of 20 and nolap=TRUE. The nolap=TRUE option prevented overlapping paired-end mates from contributing duplicate counts at the same base. CpGs with coverage above the 99.9th percentile within each sample were excluded, and coverage was normalized to the sample median. Complementary-strand CpG calls were combined using destrand=TRUE.

Methylation data were unified across the participants included in each analysis. A CpG entered differential methylation testing only if it met the 10-read minimum coverage requirement in every participant included in that analysis. Unification was performed separately within each family and for analyses involving the complete cohort; family-specific testable CpG sets were therefore allowed to differ from one another and from the CpG set available across all 17 participants. Differential methylation was assessed using the logistic regression framework implemented in methylKit, with model specifications described below. Multiple-testing correction was performed using the SLIM method. CpGs were considered significant at q<0.01 and an absolute weighted methylation difference of at least 25%.

Significant CpGs were annotated to hg19 RefSeq transcripts and CpG island features using genomation (43). Genomic coordinates were used to assign transcript region, distance to the nearest transcription start site, and CpG island or shore status. CpGs initially annotated to uncharacterised transcripts were additionally examined against NCBI RefSeq gene boundaries.

### Assessment of sequencing run concordance

Sequencing-run concordance was assessed before concatenation of the Run 1 and Run 2 reads. Methylation profiles from the independently processed sequencing datasets were analysed separately using the same minimum coverage threshold of 10 reads. CpGs with coverage above the 99.9th percentile within each dataset were excluded, coverage was normalised to the sample median, and complementary-strand CpG calls were combined during unification. CpGs on chromosomes X and Y and autosomal CpGs with zero variance across the sequencing datasets were excluded before principal component analysis. No mitochondrial CpGs remained after filtering.

Principal component analysis was performed using centred, unscaled methylation percentages from the remaining variable autosomal CpGs. The analysis included the independently processed sequencing datasets from all 17 biological samples, comprising 16 samples represented in both sequencing runs and one sample represented only in Run 1. Concordance between paired Run 1 and Run 2 profiles was assessed by comparing scores for PCs 1–5 using paired Wilcoxon signed-rank tests. P values for the five principal-component comparisons were adjusted using the Benjamini–Hochberg procedure.

As a complementary unsupervised assessment, hierarchical clustering was performed across the independently processed sequencing datasets using correlation distance and Ward linkage. Both sequencing runs were generated on the same sequencing platform with identical read lengths.

### Global methylation structure and donor variable analysis

Global methylation structure was assessed by principal component analysis using methylation percentages from all 17 participants. CpGs on chromosomes X and Y were excluded, as were autosomal CpGs with zero between-participant variance. No mitochondrial CpGs remained after coverage filtering and unification. Principal component analysis was performed on the resulting 571,676 variable autosomal CpGs after centring methylation percentages without scaling.

Associations of participant characteristics with PCs 1–5 were examined separately. Age at recruitment and BMI were assessed using Spearman rank correlation, with asymptotic P values used because of tied observations. T1D status, family membership, sex, generation and HLA class II risk-haplotype dosage, classified as zero, one or two risk haplotypes, were assessed using Kruskal–Wallis tests. Benjamini–Hochberg correction was applied separately to the five age tests, the five BMI tests and the 25 tests involving the five categorical variables across PCs 1–5.

Following the univariable analysis, PC2 was examined in a secondary multivariable linear model because BMI showed an association with this component after multiple-testing correction. The model included BMI, age at recruitment, sex, T1D status and family membership. The contribution of each predictor, including the overall family effect, was evaluated using partial F tests. Given the cohort size, these analyses were treated as exploratory assessments of global methylation structure rather than formal adjustment of the primary within-family differential methylation analysis.

### Within-family differential methylation and cross-family prioritisation

Each pedigree was analysed independently, comparing participants with T1D with their unaffected relatives. Coverage filtering, normalisation and unification were performed separately within each family, so a CpG was tested only when it satisfied the minimum coverage requirement in every participant from that pedigree. The resulting family-specific CpG sets were not required to be identical across the three families.

Two differential methylation models were fitted separately within each pedigree. The first model compared T1D and unaffected relatives without participant-level covariates. The second included age at recruitment as a continuous covariate and sex as a categorical covariate. Differential methylation was estimated using methylKit::calculateDiffMeth, with methylation differences defined as T1D minus unaffected methylation. CpGs meeting q<0.01 and an absolute weighted methylation difference of at least 25% were retained from each family-specific analysis.

Cross-family recurrence was evaluated separately for the unadjusted and age- and sex-adjusted analyses. Family-specific results were matched by hg19 chromosome, genomic coordinate and strand. A CpG was classified as recurrent only if it independently met the significance and effect-size criteria in all three pedigrees and showed the same direction of methylation difference in each family. The unadjusted cross-family analysis defined the initial recurrent CpG set. CpGs from this set that retained the significance, effect-size and directional criteria after adjustment for age and sex were prioritised for genomic annotation, transcriptomic comparison and pyrosequencing assessment. The participant-level design matrices for the adjusted models were full rank in all three families (rank = 4). With six, seven and four participants in Families A, B and C, respectively, this left two, three and zero participant-level residual degrees of freedom. The Family C model was therefore saturated. The adjusted within-family analyses were used for candidate prioritisation rather than interpreted as providing stable covariate-adjusted effect estimates.

For the six prioritised loci, candidate-level diagnostic refits were performed using the original methylated and unmethylated read counts and the same participant-level binomial model specification. Model diagnostics included assessment of coefficient finiteness, numerical convergence and fitted probabilities approaching the boundaries of 0 or 1.

For comparison with the single-run dataset, Run 1 was analysed using the same unadjusted within-family model, filtering criteria and cross-family recurrence definition.

### Locus-specific coverage assessment of prioritised CpGs

Raw, non-normalised read depth was extracted at the exact hg19 coordinates of the six prioritised CpGs from the independently processed Run 1, Run 2 and merged datasets. Coverage was evaluated separately for each locus and family. A locus was defined as coverage-complete within a family only when every participant in that pedigree had at least 10 reads at the CpG.

Coverage completeness was evaluated independently of differential methylation significance. A coverage-complete locus was not considered recurrent unless it also met q<0.01 and an absolute weighted methylation difference of at least 25% independently in all three families, with concordant methylation direction across the pedigrees. For the merged dataset, raw read depth was summarised as means and ranges for participants with T1D and unaffected relatives, both across the cohort and within each family.

### Cohort-wide covariate and family-membership sensitivity analyses

The six prioritised CpGs were subsequently evaluated in cohort-wide sensitivity analyses using all 17 participants as individual samples. These analyses were performed after candidate prioritisation and were not used for candidate selection. Their purpose was to assess whether the six loci retained the prespecified methylation differences after accounting for additional participant characteristics and average methylation differences among the three families.

Nine genome-wide methylKit models were fitted: an unadjusted model; models adjusted individually for age at recruitment, sex or BMI; models adjusted jointly for age and sex or for age, sex and BMI; and three models incorporating family membership as a three-level categorical fixed covariate, either alone, with age and sex, or with age, sex and BMI. Differential methylation was assessed using the methylKit logistic-regression framework with a chi-squared test, weighted methylation differences and SLIM multiple-testing adjustment. Each model was fitted across the 630,081 CpGs available in the complete cohort, after which results for the six prioritised loci were extracted. A prioritised locus was considered retained when it continued to satisfy q<0.01 and |Δβ|≥25%.

Family membership was modelled as a fixed categorical covariate to account for average methylation differences among Families A, B and C. This specification does not model pedigree structure or within-family correlation explicitly. The cohort-wide models were therefore interpreted as post-prioritisation sensitivity analyses and not as substitutes for the within-family screening and prioritisation strategy.

### Pyrosequencing assessment

Targeted pyrosequencing was used as an orthogonal assay to quantify methylation at the six prioritised CpG loci and to examine within-family methylation patterns. Genomic DNA was bisulfite converted using the EpiTect Fast 96 Bisulfite Conversion Kit (Qiagen) according to the manufacturer’s protocol. Locus-specific PCR amplification was performed using custom PyroMark primers and PyroMark PCR Master Mix (Qiagen); primer sequences and target regions are provided in **Supplementary Table 3**. Pyrosequencing was performed on a PyroMark Q24 instrument (Qiagen), and methylation was quantified as the percentage of methylated cytosine at the targeted CpG. Assays were designed for all six prioritised loci. Only assays yielding reproducible quantitative methylation measurements were included in subsequent statistical and segregation analyses.

### Statistical analysis of pyrosequencing methylation

Pyrosequencing methylation percentages were analysed as continuous dependent variables at loci that yielded reproducible quantitative measurements. Associations with T1D status were first examined using sequential linear models. The unadjusted model included T1D status alone, followed by models adjusting separately for age at recruitment, sex, BMI, HLA class II risk-haplotype dosage or family membership. A further model included T1D status together with age, sex, BMI and HLA risk dosage. HLA risk dosage was coded as 0, 1 or 2 according to the haplotype classification defined above and reported in **Supplementary Table 2**.

Joint donor-level models included T1D status, age at recruitment, sex, BMI and HLA risk dosage simultaneously. Family structure was represented in two ways: family membership was entered as a categorical fixed effect in the principal model and as a random intercept in a complementary mixed-effects model. The family-fixed specification was treated as the principal analysis because only three pedigrees were available. Benjamini–Hochberg correction was applied across the three pyrosequenced loci separately for each predictor and model specification.

Model adequacy was assessed by examining model-matrix rank, variance inflation factors, residual distributions, leverage and Cook’s distance. Random-effect variance and boundary estimates were additionally examined for the mixed-effects models. Leave-one-participant-out analysis was performed for the principal family-fixed *MYRIP* model to assess the stability of the HLA risk-dosage and sex coefficients.

Disease duration was calculated as age at recruitment minus age at T1D diagnosis and analysed only among participants with T1D. Disease duration was examined first alone and then with adjustment for HLA risk dosage. Age and disease duration were not included in the same model because of their substantial collinearity. Linear models were fitted in R v4.4.3. Linear mixed-effects models were fitted by maximum likelihood using lme4, with P values obtained from asymptotic Wald tests. Variance inflation factors were calculated using car v3.1.5.

### Comparison with public T1D transcriptomic data and enrichment analysis

To examine the biological context of the recurrent methylation loci, genes annotated to the six prioritised recurrent CpGs were compared with differential-expression results from three public T1D transcriptomic datasets: E-MEXP-1140, comprising pancreatic tissue and purified islets (44); GSE33440, comprising peripheral-blood CD14-positive monocytes (45); and GSE72492, comprising pancreatic tissue (46). CpG-to-gene assignments were based on RefSeq positional annotation and were not assumed to indicate regulation of the annotated transcript by the corresponding CpG.

Differential expression was analysed using limma v3.42.2 (47), with significance defined as a Benjamini–Hochberg false discovery rate below 0.05. Probe annotations were mapped to gene symbols, and duplicate gene symbols were collapsed after differential-expression testing so that each gene contributed once to the differentially expressed gene set.

Enrichment of genes annotated to the recurrent CpGs among differentially expressed genes was evaluated separately for each transcriptomic dataset using an upper-tail hypergeometric test. A fixed reference universe of 20,000 genes was used for all three tests. *LINC00701* was not represented on the expression platforms and was excluded from the enrichment analysis, leaving five testable candidate genes. The expected overlap was calculated from the proportion of the 20,000-gene reference universe classified as differentially expressed in each dataset. Methylation direction was not incorporated into the enrichment calculation; methylation and expression directions were compared separately as a descriptive analysis.

## Results

### Combined sequencing runs increase coverage and expand the analysable CpG landscape

Bisulfite conversion efficiency ranged from 99.6% to 99.7% across the 17 participants, and mapping efficiency ranged from 85.6% to 86.3% (**Supplementary Table 4**). Run 1 yielded 85,922 analysable CpGs at a mean depth of 10.6× across all 17 participants. Among the 16 participants who were resequenced, Run 2 yielded 45,727 CpGs at a mean depth of 9.2×; I_2_C was not resequenced because Run 1 coverage was already sufficient at 17.7×. After merging, 630,081 CpGs were analysable at a mean depth of 19.0×, with per-sample coverage ranging from 14.1× to 25.9× (**Table 1** and **2**). The merged dataset therefore substantially expanded the common CpG set available for analysis relative to either run alone. This increase reflects the requirement for at least 10 reads in every participant, because merging increased locus-specific depth sufficiently for many CpGs that fell below this threshold in one or more participants in either run alone.

### Concordance between sequencing runs

After filtering, 27,227 variable autosomal CpGs were available for comparison across the 33 independently processed sequencing datasets, representing 16 paired Run 1 and Run 2 samples and one sample sequenced once. Paired datasets from the same biological sample occupied similar positions in principal component space, with no systematic separation by sequencing run (**Supplementary Figure 1**). PC1 and PC2 explained 9.3% and 7.2% of the variance, respectively.

Paired Wilcoxon signed-rank tests identified no significant Run 1 versus Run 2 differences in PCs 1–5 after Benjamini–Hochberg correction (adjusted P = 0.457, 0.457, 0.856, 0.457 and 0.457, respectively). Hierarchical clustering similarly grouped each Run 1 dataset most closely with its corresponding Run 2 dataset rather than by sequencing run, while the single-run sample I_2_C clustered within the Family C branch (**Supplementary Figure 2**). These analyses did not suggest a systematic sequencing-run effect on the major sources of methylation variation.

### Global methylation structure is associated with family membership

Principal component analysis of 571,676 variable autosomal CpGs across all 17 participants showed clear family-related structure on the PC1–PC2 projection, with no clear visual separation by T1D status (**Figure 2**). PC1–PC5 explained 11.0%, 8.8%, 7.5%, 7.0% and 6.8% of the variance, respectively. Family membership was associated with PC1 and PC2 after multiple-testing correction (Kruskal–Wallis H = 11.555, FDR-adjusted P = 0.026; H = 14.020, FDR-adjusted P = 0.023, respectively). BMI was associated with PC2 in the univariable analysis (Spearman ρ=−0.725, FDR-adjusted P = 0.005), T1D status with PC3 (H = 7.259, FDR-adjusted P = 0.044), and generation with PC5 (H = 9.091, FDR-adjusted P = 0.026). Age at recruitment, sex and HLA class II risk-haplotype dosage were not associated with PCs 1–5 after correction (**Supplementary Table 5**). In the multivariable PC2 model including BMI, age, sex, T1D status and family, the BMI association was attenuated (P = 0.520), whereas the overall family effect remained significant (P = 4.75×10^−5^; **Supplementary Table 6**).

**Figure 2:**
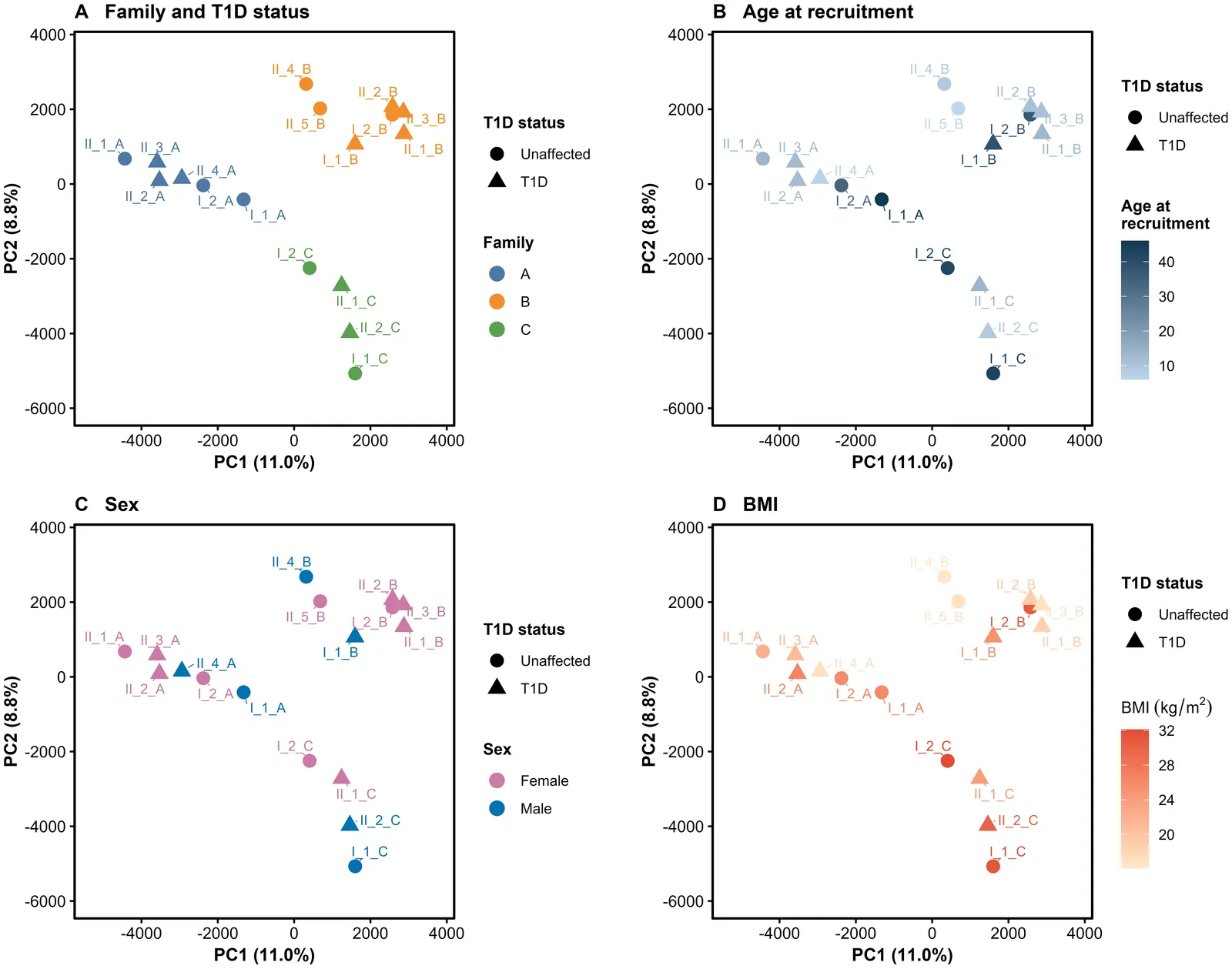
Principal component analysis of global methylation profiles in the familial T1D cohort. Principal component analysis was performed using methylation percentages at 571,676 variable autosomal CpG sites across all 17 participants. All panels show the same PC1–PC2 projection, with the percentage of variance explained shown on each axis. Participant pedigree identifiers are shown adjacent to each point. **(A)** Point colour denotes family. **(B)** Point colour denotes age at recruitment. **(C)** Point colour denotes sex. **(D)** Point colour denotes BMI. Point shape denotes T1D status in all panels, with circles representing unaffected participants and triangles representing participants with T1D. Complete association statistics for PCs 1–5 are provided in **Supplementary Table 5**.

### Cross-family recurrence before and after age and sex adjustment

The family-specific testable sets comprised 792,683 CpGs in Family A, 1,323,784 in Family B and 1,220,037 in Family C. In the unadjusted analyses, 9,138, 17,411 and 18,314 CpGs, respectively, met q<0.01 and |Δβ|≥25%. Eighty-five CpG coordinates met these criteria in all three families, of which 63 showed discordant methylation direction and were excluded. The remaining 22 CpGs showed concordant direction across all three pedigrees, comprising 10 hypermethylated and 12 hypomethylated sites in T1D (**Figure 3A**; **Supplementary Table 7**).

**Figure 3:**
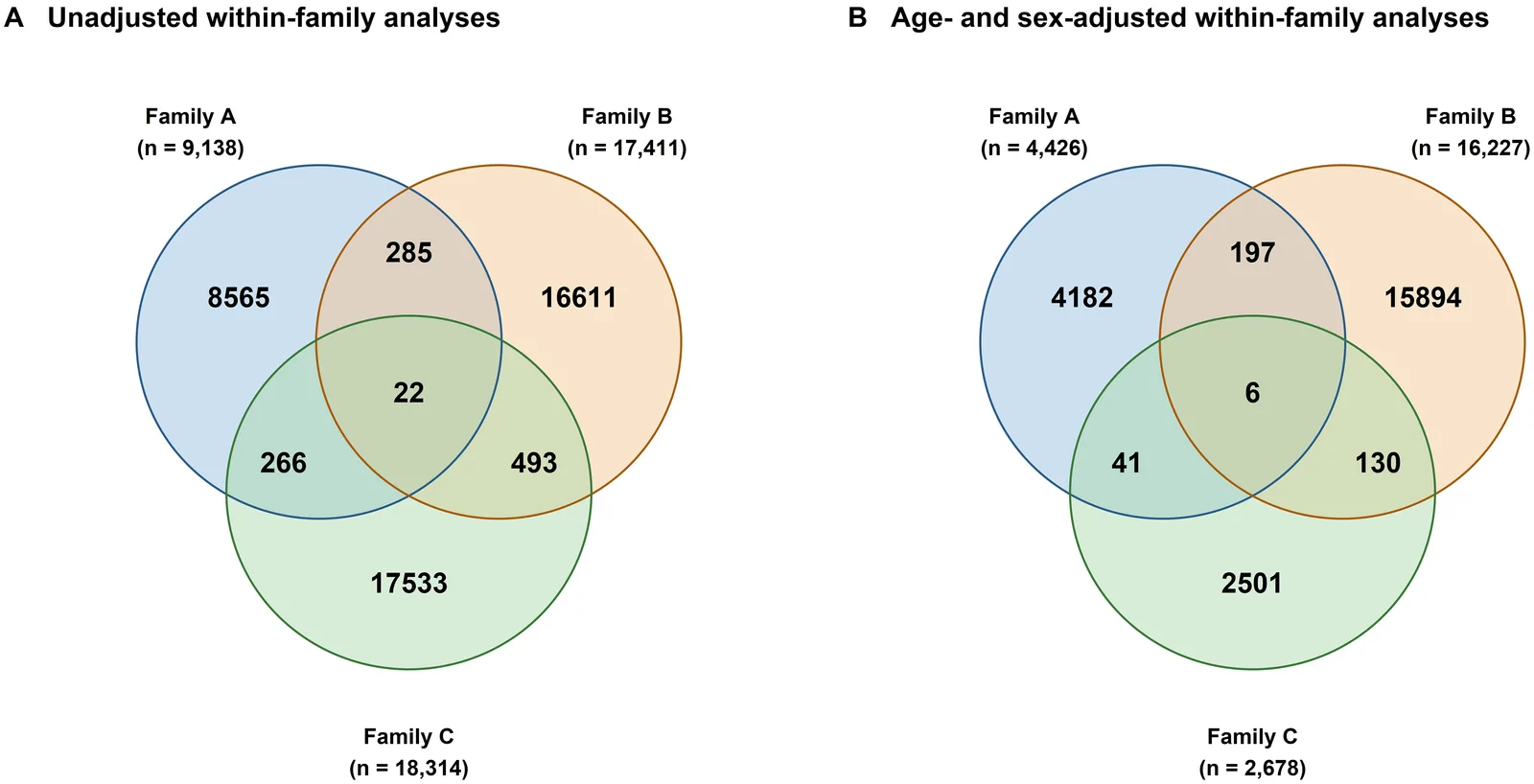
Cross-family recurrence of unadjusted and age- and sex-adjusted differentially methylated CpG sites. Venn diagrams show CpG sites meeting q<0.01 and an absolute weighted methylation difference of at least 25% in affected versus unaffected relatives within each family. Panel **(A)** presents the unadjusted within-family analyses, which identified 9,138, 17,411 and 18,314 differentially methylated CpGs in Families A, B and C, respectively. Twenty-two CpGs were significant in all three families and showed concordant methylation direction. Panel **(B)** presents the within-family analyses adjusted for age at recruitment and sex, which identified 4,426, 16,227 and 2,678 differentially methylated CpGs in Families A, B and C, respectively. Six CpGs retained the prespecified criteria in all three families with concordant direction and were prioritised for subsequent analyses. Pairwise and three-family overlap counts include only CpGs showing the same direction of methylation change in the families represented. Positive methylation differences indicate hypermethylation in T1D, whereas negative differences indicate hypomethylation.

After adjustment for age at recruitment and sex within each family, 4,426, 16,227 and 2,678 CpGs met the same criteria in Families A, B and C, respectively. Fourteen CpG coordinates were common to all three families; eight showed discordant direction, leaving six CpGs that retained the significance, effect-size and directional criteria and were prioritised for subsequent analyses (**Figure 3B**; **Table 3**; **Supplementary Tables S7**, **S8**). All six were already present in the unadjusted 22-CpG recurrent set. They mapped to *EIF1B-AS1/MYRIP*, *LOC105377777/DLGAP2*, *ULK4*, *MTCL1*, *LINC00701* and *CCNG1* and were carried forward for genomic annotation, transcriptomic comparison and pyrosequencing assessment.

**Table 3:** Prioritised recurrent CpG sites retained after age and sex adjustment and expression profiles of their annotated genes in public T1D transcriptomic datasets.

Gene	Methylation direction	CpG Site	Functional region	Expression dataset	log2FC (T1D vs control)	Adjusted P (FDR)	Expression direction
*MYRIP*	Hypomethylated in T1D	chr3:40284731	Intron	E-MEXP-1140	3.44	1.28×10^−5^	Upregulated in T1D
*DLGAP2*	Hypomethylated in T1D	chr8:817092	Intron	GSE33440	1.03	3.93×10^−3^	Upregulated in T1D
*ULK4*	Hypermethylated in T1D	chr3:41997059	Intron	E-MEXP-1140	1.94	6.23×10^−3^	Upregulated in T1D
*MTCL1*	Hypermethylated in T1D	chr18:8802954	Intron	E-MEXP-1140	−2.05	1.27×10^−3^	Downregulated in T1D
*LINC00701*	Hypomethylated in T1D	chr10:2357121	Shores, promoter, intron	—	—	—	—
*CCNG1*	Hypomethylated in T1D	chr5:162758336	—	E-MEXP-1140	4.40	3.90×10^−7^	Upregulated in T1D
				GSE72492	7.23	2.77×10^−4^	

Hypomethylation indicates lower methylation and hypermethylation indicates higher methylation in participants with T1D relative to unaffected relatives within the same family. log2FC: limma contrast T1D vs control; positive=higher in T1D. Adjusted P: Benjamini–Hochberg FDR from limma. CpGs at chr3:40284731 and chr8:817092 lie within introns shared by EIF1B-AS1/MYRIP and LOC105377777/DLGAP2; protein-coding gene names are used. (—)=not available..

Candidate-level diagnostic refitting of the six prioritised loci yielded finite coefficients and formal numerical convergence for all 18 family-by-locus models. However, fitted probabilities approached 0 or 1 for *MYRIP* and *ULK4* in Family B, and the saturated Family C models produced large coefficient estimates despite formal convergence. These diagnostics further supported using the adjusted within-family analyses for candidate prioritisation rather than interpreting them as stable covariate-adjusted effect estimates.

### Coverage assessment of the six prioritised CpG sites across separate and merged sequencing runs

Locus-specific coverage was assessed at the six prioritised CpGs in the separate and merged datasets. In Run 1, 6 of 18 locus-by-family combinations were coverage-complete, compared with 2 of 18 in Run 2 and 18 of 18 after merging (**Supplementary Table 9**). *MYRIP* was the only prioritised locus with complete coverage across all three families in Run 1 and was one of the two recurrent CpGs identified from Run 1 alone. No locus was coverage-complete across all three families in Run 2; I_2_C was not resequenced because sufficient coverage had been obtained in Run 1.

After merging, all six loci had at least 10 reads in every participant. Coverage ranges overlapped between affected and unaffected participants, with mean T1D-to-unaffected coverage ratios ranging from 0.79 to 1.30. Mean depth was higher in T1D at *DLGAP2*, *ULK4* and *LINC00701* and lower at *MYRIP*, *MTCL1* and *CCNG1*, showing no consistent direction of coverage imbalance by disease status.

### Sensitivity to coordinate-based deduplication

Coordinate-based deduplication reduced mean coverage from 19.0× in the primary analysis to 10.0 ± 2.1× and reduced the common CpG set meeting the 10-read threshold across all 17 participants from 630,081 to 129,174. The family-specific testable sets comprised 167,379 CpGs in Family A, 571,734 in Family B and 472,899 in Family C. In the unadjusted deduplicated analyses, 152, 2,163 and 248 CpGs, respectively, met q<0.01 and |Δβ|≥25%, but no CpG retained concordant three-family recurrence.

Candidate-level examination showed that loss of three-family recurrence reflected several mechanisms. *DLGAP2*, *LINC00701* and *CCNG1* were no longer coverage-complete in any family, while *ULK4* and *MTCL1* were not coverage-complete in Family A. *MYRIP* remained coverage-complete in all three families; in Family A, however, its weighted methylation difference decreased to −17.1% and did not retain statistical significance (q=0.653), whereas both the effect-size and significance criteria were retained in Families B and C. *ULK4* retained weighted methylation differences above 25% in Families B and C (+35.0% and +49.1%, respectively), but did not retain q<0.01 in either family (q=0.020 and q=0.129). *MTCL1* retained both criteria in Family B (+37.5%, q=8.67×10^−5^), whereas in Family C its weighted methylation difference decreased to +23.7% and was not significant (q=0.625). No evaluable comparison showed reversal of methylation direction. Thus, failure of the three-family recurrence criterion after deduplication reflected a combination of reduced coverage completeness, loss of statistical significance and, for *MYRIP* in Family A and *MTCL1* in Family C, effect-size attenuation (**Supplementary Table 18**).

### Comparison with run 1 and the previous study

In the unadjusted Run 1 within-family analyses, 628, 3,831 and 3,722 CpGs met q<0.01 and |Δβ|≥25% in Families A, B and C, respectively (**Supplementary Table 10**). Two CpGs met these criteria with concordant hypomethylation across all three families: chr3:40284731 (*EIF1B-AS1/MYRIP*) and chr8:95544723 (*VIRMA*) (**Supplementary Figure 3**). Five of the six loci prioritised from the combined dataset lacked complete coverage in at least one family in Run 1; *MYRIP* was the exception and was retained in the Run 1 intersection. The chr3:40284731 *EIF1B-AS1/MYRIP* locus was also identified in our previous pooled analysis (34), with hypomethylation in affected participants (meth.diff=−43.8, q=5.9×10^−26^).

### Cohort-wide sensitivity analyses of the six prioritised loci

Cohort-wide sensitivity analyses using all 17 participants showed that all six prioritised CpGs retained q<0.01 and |Δβ|≥25% across all nine model specifications, including models incorporating participant characteristics and family membership (**Supplementary Table 11**). Methylation direction remained unchanged: *EIF1B-AS1/MYRIP*, *LOC105377777/DLGAP2*, *LINC00701* and *CCNG1* were hypomethylated in T1D, whereas *ULK4* and *MTCL1* were hypermethylated.

In the fully adjusted model, q values ranged from 1.10×10^−38^ for *EIF1B-AS1/MYRIP* to 1.92×10^−13^ for *LINC00701*. Across the nine genome-wide models, 608 to 630 CpGs met the same significance and effect-size criteria.

### Biological context from public t1d transcriptomic datasets

Five of the six prioritised CpGs were annotated to protein-coding genes represented in the public transcriptomic datasets; *LINC00701* was not represented. *MYRIP*, *ULK4*, *MTCL1* and *CCNG1* were differentially expressed in E-MEXP-1140, *DLGAP2* in GSE33440, and *CCNG1* also in GSE72492 (**Table 3**). Hypergeometric testing did not show enrichment of the five candidate genes beyond chance expectation in any dataset: four of five were observed in E-MEXP-1140 compared with 1.93 expected (P = 0.077), one of five in GSE33440 compared with 0.94 expected (P = 0.647), and one of five in GSE72492 compared with 0.20 expected (P = 0.184; **Supplementary Table 12**).

In directional comparisons, *MYRIP*, *DLGAP2* and *CCNG1* were hypomethylated and upregulated in T1D, whereas *MTCL1* was hypermethylated and downregulated; *ULK4* was both hypermethylated and upregulated. These comparisons were descriptive because methylation and expression were measured in different participants and, for some datasets, different tissues or cell populations, and they do not establish regulation of the annotated gene by the corresponding CpG.

### Pyrosequencing assessment and within-family segregation

Pyrosequencing yielded reliable quantitative data for three of the six prioritised loci: *MYRIP*, *ULK4* and *MTCL1* (**Figure 4**).

**Figure 4:**
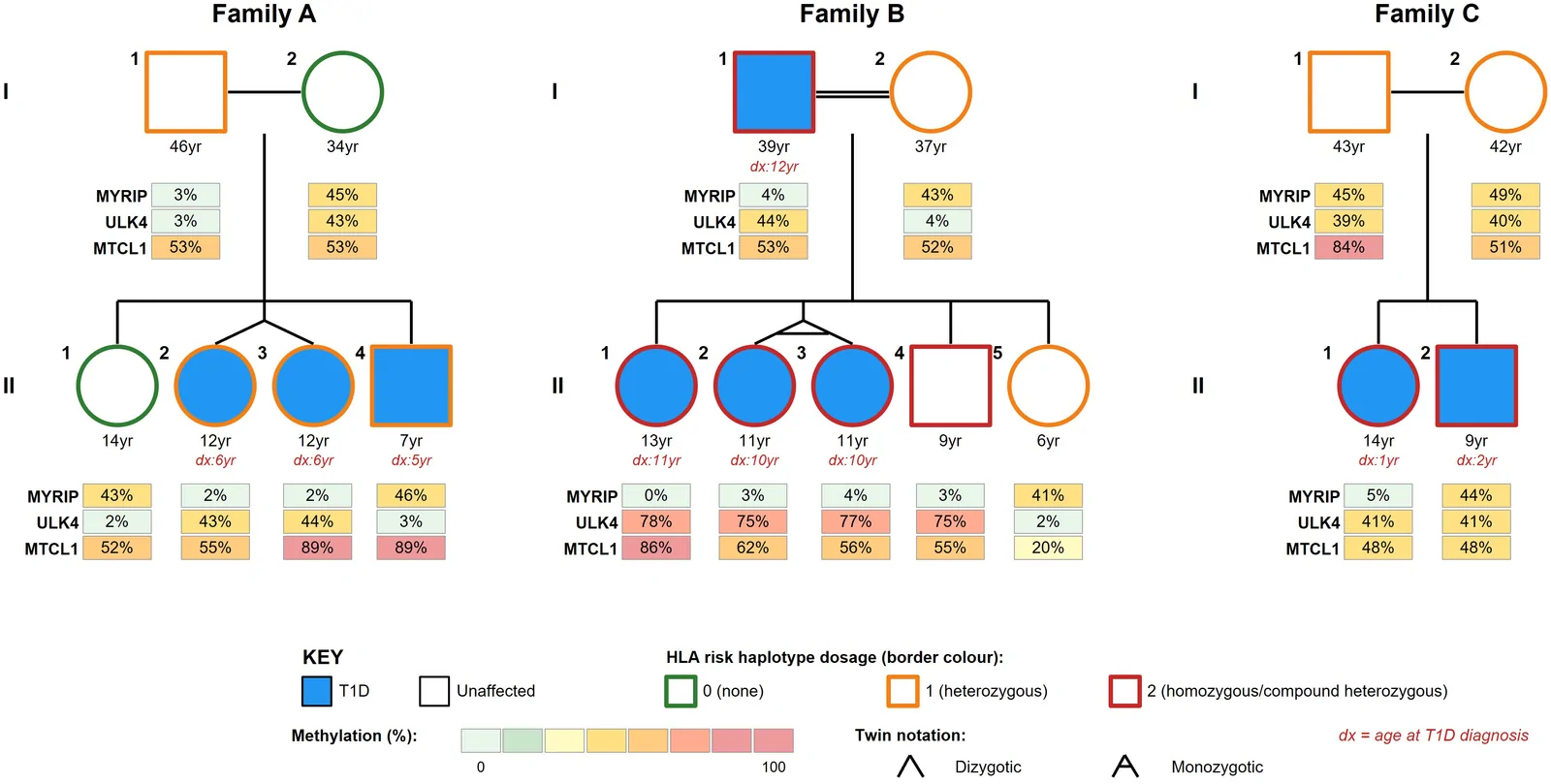
Pedigrees of Families A, B, and C with pyrosequencing-derived methylation values (%) at *MYRIP*, *ULK4*, and *MTCL1* for each individual. Circles represent females and squares represent males. Blue-filled symbols indicate T1D-affected individuals; unfilled symbols indicate unaffected individuals. Border colour denotes HLA class II risk haplotype dosage: green=zero high-risk haplotypes, orange=one high-risk haplotype (heterozygous), red=two high-risk haplotypes (homozygous DR3-DQ2 or compound heterozygous DR3-DQ2/DR4-DQ8). Methylation values are displayed from top to bottom for *MYRIP*, *ULK4* and *MTCL1*. Methylation values are displayed as colour-coded cells on a gradient from green (0%) through yellow and orange to red (100%). Twin pairs are indicated by a shared connector: an angled connector (∧) for dizygotic twins and a horizontal bar connector for monozygotic twins. Roman numerals denote generations (I=parents, II=children). Ages at time of recruitment are shown below each individual; age at T1D diagnosis (*dx*) is indicated in red where applicable. Full HLA class II genotypes are provided in **Supplementary Table 2**.

At chr3:40284731 (*EIF1B-AS1/MYRIP*), methylation showed a bimodal distribution, with low (0 to 5%; n=9) and partial (41 to 49%; n=8) classes and no intermediate values. Affected and unaffected relatives were represented in both classes. In Family A, the affected dizygotic twins II_2_A and II_3_A shared the low-methylation state with their unaffected father, whereas affected II_4_A and unaffected II_1_A shared the partial state with their unaffected mother. In Family B, affected father I_1_B had 4% methylation and unaffected mother I_2_B had 43%; four of five offspring, including the affected monozygotic twins II_2_B and II_3_B, were in the low class, whereas unaffected II_5_B was in the partial class. In Family C, affected II_1_C and II_2_C occupied different classes. *MYRIP* methylation therefore did not segregate with T1D status at the individual-level.

At chr3:41997059 (*ULK4*), methylation showed low (0 to 4%; n=5), intermediate (39 to 44%; n=8) and high (75 to 78%; n=4) classes. In Family A, affected and unaffected participants occurred in both the low and intermediate classes. In Family B, four of five offspring were in the high class, including the unaffected II_4_B alongside three affected siblings. In Family C, both parents and both affected offspring had intermediate methylation values. *ULK4* methylation therefore did not segregate consistently with disease status.

At chr18:8802954 (*MTCL1*), methylation varied continuously from 20 to 89%. In Family A, affected II_3_A and II_4_A had methylation values of 89%, whereas affected II_2_A had 55%, close to the parental values of 53%. In Family B, methylation varied widely among the offspring, including both affected and unaffected individuals. In Family C, the unaffected father had a higher methylation value than either affected offspring. *MTCL1* therefore showed incomplete segregation with T1D status.

Across all three loci, affected and unaffected relatives occupied overlapping methylation classes or ranges, indicating incomplete within-family segregation.

### Assessment of local genetic variation

The available WES gVCFs did not provide informative genotyping at any of the six prioritised CpGs. At each locus, nine of 17 participants had a reference block spanning the CpG with zero read depth, while the remaining eight had no record spanning the position. No explicit SNP or indel call was present within ±500 bp of any of the six CpGs. Because these noncoding intervals were not informatively captured by WES, the absence of variant calls does not exclude local sequence variation.

### HLA class II genotyping

HLA class II genotypes were determined for all 17 participants (**Supplementary Table 2**). Clinical-grade typing was also available for 13 participants, including one participant with a duplicate clinical-grade measurement, giving 42 locus comparisons with the exome-derived calls. Agreement was complete at first-field resolution (42/42) and 88.1% at second-field resolution (37/42); none of the second-field differences altered HLA risk-haplotype classification (**Supplementary Table 13**). All offspring carried haplotypes consistent with Mendelian transmission in the three pedigrees. Two participants carried no defined high-risk haplotype, eight carried one, and seven carried two. T1D was present in 0 of 2, 3 of 8 and 6 of 7 participants in the zero-, one- and two-risk groups, respectively. Among the seven participants carrying two high-risk haplotypes, five were DR3-DQ2 homozygotes and two were DR3-DQ2/DR4-DQ8 compound heterozygotes. The only unaffected participant in this group, II_4_B, was 9 years old at recruitment and carried the same DR3-DQ2 homozygous genotype as the affected monozygotic twins II_2_B and II_3_B, with similar methylation values at all three pyrosequenced loci (**Figure 4**). Allele counts and cohort frequencies are provided in **Supplementary Table 14**.

### Associations of pyrosequencing methylation with donor characteristics

Pyrosequencing methylation at *MYRIP*, *ULK4* and *MTCL1* was first assessed in sequential models of T1D status (**Supplementary Table 15**). *MYRIP* methylation was nominally lower in participants with T1D in the unadjusted model (β=−21.78 percentage points, 95% CI −41.40 to −2.16, P = 0.032), but this association did not survive correction across the three loci (q=0.096). Adjustment separately for age, sex, BMI or family produced estimates of similar direction and magnitude, whereas inclusion of HLA risk-haplotype dosage reduced the T1D-status coefficient to −11.33 percentage points (P = 0.315). No T1D-status association at *MYRIP*, *ULK4* or *MTCL1* reached q<0.05 in any sequential model.

In the fully adjusted family-fixed model, the *MYRIP* T1D-status coefficient changed from −21.78 percentage points in the unadjusted model to +15.94 percentage points and remained non-significant after multiple-testing correction (q=0.277). Higher HLA risk-haplotype dosage was associated with lower *MYRIP* methylation in the fully adjusted models (**Supplementary Table 16**), with an estimated change of −56.77 percentage points per additional risk haplotype in the family-fixed model (95% CI −82.37 to −31.18, P = 0.0007, q=0.002) and −51.99 percentage points in the random-intercept model (q<0.001). In leave-one-participant-out analyses, the HLA risk-dosage coefficient remained negative in all 17 iterations (β range, −67.26 to −41.85; all P<0.05).

Male sex was associated with higher *MYRIP* methylation in the fully adjusted models (family-fixed β=+22.05, q=0.032; random-intercept β=+20.60, q<0.001), but this association was not evident in the simpler sex-adjusted model and the coefficient was less stable in leave-one-participant-out analyses (**Supplementary Table 16**). Age and BMI were not associated with methylation at any of the three loci after correction. Associations of *ULK4* methylation with HLA dosage and *MTCL1* methylation with T1D status were observed only in random-intercept models with near-zero family-variance estimates and were not supported by the principal family-fixed models.

Among the nine participants with T1D, no association between disease duration and methylation survived correction at any locus (**Supplementary Table 17**). Age and disease duration were strongly correlated (r=0.910).

## Discussion

This study identified 22 CpGs with recurrent concordant methylation differences across three pedigrees in the unadjusted within-family screen, of which six were prioritised after retaining the prespecified criteria following age and sex adjustment. Pyrosequencing at *MYRIP*, *ULK4* and *MTCL1* revealed structured methylation variation, but affected and unaffected relatives occupied overlapping states or ranges. Recurrence across pedigrees therefore did not translate into deterministic segregation within families. The recurrent differences do not establish whether methylation precedes, follows, or contributes to T1D. Their incomplete segregation within the pedigrees argues against a simply inherited methylation state.

The present analysis extends our previous pooled comparison of the same families (34) by asking whether methylation differences recur independently within each pedigree. The chr3:40284731 locus at *EIF1B-AS1/MYRIP* was recovered in both studies with hypomethylation in affected participants despite the different analytical designs.

### Sensitivity analyses of the prioritised recurrent loci

Adjustment for age and sex reduced the recurrent set from 22 to six CpGs, all of which were already present in the unadjusted intersection. The adjusted analysis therefore served as a prioritisation step applied to an existing cross-family signal rather than identifying a separate set of loci, and the six retained CpGs were treated as prioritised recurrent candidates. The participant-level design matrices were full rank, but the small pedigree sizes provided limited residual information: Families A and B retained two and three participant-level residual degrees of freedom, respectively, whereas the four-participant Family C model was saturated. The adjusted within-family estimates, particularly for Family C, were therefore not interpreted as stable age- and sex-adjusted effects. The loss of the remaining loci after adjustment should not be interpreted as evidence that their unadjusted associations were necessarily spurious. With only four to seven participants per pedigree, covariate adjustment can substantially alter effect estimates and precision, particularly where age, generation and disease status are correlated. The larger number of differentially methylated CpG sites in Family B may partly reflect its greater number of participants, broader testable CpG space and different age, generation and phenotype structure. Absolute numbers of differentially methylated CpGs were therefore not interpreted as evidence of greater methylation burden in Family B; prioritisation instead relied on cross-family recurrence with concordant direction.

The six prioritised CpGs also satisfied the prespecified effect-size and significance criteria across complementary cohort-wide sensitivity models adjusting for participant characteristics and family membership. Their methylation directions remained unchanged across these model specifications. These analyses do not, however, model kinship explicitly because family was represented as a categorical fixed effect. They therefore provide post-selection sensitivity evidence for the six prioritised loci rather than independent replication.

Combining the two sequencing runs increased the CpG space available for cross-family testing. Five of the six prioritised loci lacked complete family-level coverage in Run 1, whereas all six were coverage-complete after merging. *MYRIP* alone had complete coverage across all three families in Run 1 and was already present among the two recurrent Run 1 CpGs. Coverage at the six loci showed no consistent imbalance between affected and unaffected participants, arguing against a systematic disease-associated depth effect. Coordinate-based deduplication substantially reduced sequencing depth and the CpG space available for cross-family testing. Candidate-level examination showed that loss of three-family recurrence reflected reduced coverage completeness, loss of statistical significance in several coverage-complete comparisons, and effect-size attenuation at *MYRIP* in Family A and *MTCL1* in Family C, rather than reversal of methylation direction. Because these targeted-enrichment libraries lacked unique molecular identifiers, reads sharing genomic coordinates could not be distinguished reliably as PCR-derived copies or independently captured fragments. The same indexed libraries were also sequenced in both runs, so PCR-derived molecules already present in a library could have been sampled in both datasets. Merging the runs may therefore overestimate the number of unique molecules contributing to read depth. Coordinate-based deduplication was retained as a processing sensitivity analysis rather than applied to the primary dataset.

### Incomplete segregation and locus-specific methylation architecture

Pyrosequencing revealed distinct methylation patterns at the three successfully assayed loci. The bimodal *MYRIP* distribution may reflect genetically structured methylation, local sequence variation affecting the CpG or surrounding region, or other regulatory influences. Affected and unaffected relatives occurred in both methylation states, arguing against simple segregation with T1D. The available WES data did not provide informative coverage at this locus, so local sequence variation cannot be excluded. A variant affecting the CpG or assay sequence could influence methylation measurement, whereas a cis-acting variant could generate a genuine genetically structured methylation difference. Distinguishing these possibilities would require direct genotyping together with genotype-methylation or allele-specific analyses. The trimodal distribution at *ULK4* similarly suggests structured regulation, but the observed classes do not conform to the approximately 0%, 50% and 100% pattern expected under a simple biallelic model. In Family B, four of five offspring had methylation values higher than both parents, including both affected and unaffected individuals. This pattern is not readily explained by simple additive inheritance and may reflect more complex genetic regulation, cell composition, or other trans-acting influences. By contrast, *MTCL1* showed a continuous methylation distribution, which provides little support for a discrete allelic mechanism. The modest differences at *MTCL1* and *ULK4* between the monozygotic twins further illustrate that methylation can vary despite identical genotype and concordant T1D status.

These patterns should also be interpreted in the context of the biological material. PBMC methylation represents a composite of leukocyte populations with distinct methylation profiles (48), so variation in cell composition may contribute to the observed distributions. Reference-based approaches such as Houseman and IDOL were developed for Illumina methylation arrays and have not been established for the targeted CpG content of the TruSeq Methyl Capture EPIC assay (49, 50). Requiring the same methylation direction across three pedigrees reduces the likelihood that a family-specific cell-composition difference alone generated the recurrent signal, but it cannot exclude systematic differences associated with T1D.

### HLA risk and donor-level associations

HLA risk-haplotype dosage showed a gradient with disease occurrence in these families, but individual outcomes remained non-deterministic. This was evident in Family A, where affected siblings carried different *MYRIP* methylation states and an unaffected sibling shared the partial methylation state with an affected sibling. Family B provided a complementary example: II_4_B carried the same homozygous DR3-DQ2 genotype and similar methylation values at all three pyrosequenced loci as the affected monozygotic twins, yet remained unaffected at recruitment. Because II_4_B was younger than the ages at diagnosis of the affected offspring in this pedigree, later progression cannot be excluded. Together, these observations show that neither HLA risk dosage nor the methylation states measured here were sufficient to determine disease status within the pedigrees.

The donor-level models address a different question from individual segregation. T1D-status associations with pyrosequencing methylation were sensitive to model specification and did not remain significant in the principal family-fixed models after correction. By contrast, higher HLA risk dosage was associated with lower *MYRIP* methylation after multivariable adjustment, and the coefficient remained negative in all leave-one-participant-out analyses. This association is consistent with a contribution of genetic background to *MYRIP* methylation variation, but it does not establish a direct effect of HLA genotype on the locus. The HLA-dosage coefficient should therefore be interpreted as a model-specific association within this cohort rather than as a population per-haplotype effect. HLA dosage, family membership and T1D status were correlated, and only two participants carried no defined risk haplotype. A linked familial variant or cis-acting meQTL therefore remains an alternative explanation.

The adjusted association between male sex and *MYRIP* methylation was not evident when sex was examined in a simpler model and should therefore be regarded as secondary. Age, BMI and disease duration showed no corrected association with methylation at the three pyrosequenced loci, although the small cohort limits inference about weaker effects.

### Functional relevance of recurrent loci

The recurrent loci differ substantially in the strength of their biological support. Among them, *MYRIP* has the clearest connection to pancreatic β-cell function. *MYRIP* encodes a Rab27a effector involved in tethering secretory granules to the cortical actin cytoskeleton and in organisation of the exocytic machinery (51, 52). These functions provide a plausible link between altered regulation of *MYRIP* and insulin granule release. *MYRIP* was also differentially expressed in the T1D pancreatic and islet dataset, although the present study cannot establish that methylation at chr3:40284731 regulates its expression.

The evidence for *ULK4*, *MTCL1* and *CCNG1* is more indirect. *ULK4* is a pseudokinase involved in cytoskeletal organisation and microtubule regulation (53), whereas *MTCL1* contributes to microtubule bundling and Golgi-associated microtubule organisation (54, 55). Both functions are potentially relevant to cellular organisation and vesicle trafficking, but direct evidence in pancreatic β-cells or T1D-relevant immune cells is lacking. *CCNG1* is a p53-responsive regulator of cell cycle control and stress-related survival (56, 57). Its reported involvement in cellular responses to high glucose provides biological plausibility, but this evidence derives from a non-autoimmune experimental system (58). These loci should therefore be regarded as mechanistically plausible candidates rather than established components of T1D pathogenesis.

The remaining loci have weaker functional support. The CpG at chr8:817092 lies within an intron shared by *DLGAP2* and *LOC105377777*. *DLGAP2* was emphasised for functional interpretation because methylation near the imprinted *DLGAP2* region has previously been associated with familial T1D risk in the DAISY cohort (59). However, *DLGAP2* has no established β-cell or immune function. *LINC00701* is a long intergenic non-coding RNA with little evidence linking it to immune regulation or T1D, and the biological target of methylation at this locus remains uncertain.

Comparison with public T1D transcriptomic datasets provided additional biological context but not independent validation. Five protein-coding genes annotated to the recurrent CpGs were differentially expressed in at least one dataset, but their overlap with differentially expressed genes did not exceed chance expectation. Methylation and expression were also measured in different participants and, depending on the dataset, in different tissues or cell populations. These comparisons therefore support locus-level interpretation but cannot establish methylation-dependent regulation of the annotated genes.

Most prioritised CpGs were intronic, which further limits simple interpretation of methylation direction. Unlike promoter methylation, gene body and intronic methylation can show either positive or negative relationships with transcription (30, 31). The concordant methylation and expression directions observed at several loci are therefore biologically interesting but should not be interpreted as evidence of a direct regulatory relationship.

### Strengths and limitations

A principal strength of this study is the use of three pedigrees as independent units of analysis, with recurrent CpGs required to satisfy the statistical and effect-size criteria in each family and to show the same methylation direction. The study also incorporated assessment of sequencing run concordance, locus-specific coverage, alternative read processing, cohort-wide covariate sensitivity analyses, HLA haplotype reconstruction, and orthogonal pyrosequencing. Together, these analyses allowed the recurrent signals to be examined at technical, statistical, genetic and individual-levels.

Several limitations constrain interpretation. The cohort comprised only 17 participants from three pedigrees, limiting statistical power, precluding formal statistical modelling of segregation, and restricting comprehensive modelling of within-family relatedness. The donor-level models were particularly constrained by correlation among T1D status, HLA risk dosage and family membership, and by the presence of only two participants without a defined HLA risk haplotype. Near-zero family variance estimates in some random-intercept models further limited their interpretation. The donor-level analyses should therefore be considered exploratory.

Methylation was measured in bulk PBMCs at a single time point. The study cannot distinguish methylation associated with disease susceptibility from changes related to immune cell composition, established disease, insulin treatment or other exposures. Unaffected relatives were not systematically screened for islet autoantibodies, and glucose tolerance data were not consistently available, so preclinical autoimmunity or dysglycaemia cannot be excluded. Among affected participants, disease duration was strongly correlated with age, limiting assessment of their independent effects.

Technical and validation limitations also remain. Reliable pyrosequencing was obtained for three of the six prioritised loci and was performed in the same participants used for discovery, providing orthogonal measurement rather than independent biological replication. The recurrent cross-family intersection was sensitive to coordinate-based deduplication, although deduplication substantially reduced informative coverage and duplicate origin could not be resolved in these targeted-enrichment libraries. Standard bisulfite methods do not distinguish 5-methylcytosine from 5-hydroxymethylcytosine. The available WES data did not informatively cover the six prioritised CpGs; although no explicit SNP or indel call was present within ±500 bp of these sites, local sequence variation cannot be excluded. Finally, the public transcriptomic analyses involved independent cohorts, population backgrounds not matched to the present Kuwaiti Arab cohort, and different tissues or cell populations. These differences may contribute to expression heterogeneity, and the comparisons therefore provide biological context rather than direct evidence linking CpG methylation to transcription.

### Implications and future directions

The prioritised CpGs provide candidates for investigating relationships between locus-specific methylation, HLA risk, non-HLA genetic background and T1D in larger family cohorts. Establishing whether these signals generalise will require replication in larger independent T1D kindreds, including populations outside the present Arab cohort, with longitudinal follow-up of unaffected relatives and systematic assessment of islet autoantibodies. Cell-specific methylation profiling would help distinguish disease-associated epigenetic variation from differences in leukocyte composition. Genetic and functional studies in β-cells and relevant immune cell subsets will be required to determine the mechanisms underlying the recurrent methylation patterns and whether any contribute directly to T1D-related cellular phenotypes.

## Conclusion

This study identified 22 recurrent single-CpG methylation differences with concordant direction across three Arab pedigrees in the unadjusted within-family screen, from which six loci were prioritised after retaining the prespecified criteria following age and sex adjustment. Pyrosequencing of *MYRIP*, *ULK4* and *MTCL1* revealed distinct methylation distributions, but affected and unaffected relatives overlapped, indicating incomplete segregation with T1D status. Donor-level associations were model-dependent, and the relationship between HLA risk dosage and *MYRIP* methylation remains exploratory in this small cohort. The six prioritised loci therefore represent exploratory recurrent methylation candidates. Their genetic and biological basis, temporal relationship with T1D, and reproducibility require investigation in larger independent and longitudinal family cohorts.

## Data Availability

The original targeted bisulfite sequencing data are deposited in the NCBI Sequence Read Archive under BioProject PRJNA783490.
